# The Mother’s Hand-Book

**Published:** 1891-11

**Authors:** 


THE MOTHER'S HAND-BOOK. A Practical Treatise on the Management of Children in Health and Disease. With an Appendix containing Articles on Diseases and Accidents that may suddenly happen to Grown Persons. By LEVIN J. WOOLLEN, M. D. Richmond, Va.: Everett Waddy Co. 1891.

This volume is a handsomely attired octavo on an important subject, and, no doubt, will do much good by disseminating useful information among non-professional readers, especially mothers, for whom it is more particularly intended. The title would indicate that it is a practical treatise on the management of children in health and disease, but we are of the opinion that it is not so much a practical treatise as it is a guide to the home management of children, where something must be done before the doctor comes. The fact is, no book can inform any mother how to manage her children independently of the family physician, and the further fact is, that no doctor has a right to prepare a book expecting to accomplish this impossible feat.

In the work before us will be found much valuable information written in a popular style, and certainly it may properly form part of a mother's library, but it is to be regarded more as a guide to hygienic care rather than to medical treatment. An appendix is added, which contains articles on Diseases and Accidents which may suddenly happen to grown persons. Why such an addendum has been made to the Mother's Hand-book does not appear; but here it is, and so we conclude there must be a necessity for such commingling of heterogeneous subjects. The printing of this book has been handsomely done by the Everett Waddy Co., of Richmond. Surely, if Richmond can do such beautiful work, it is an indication that she is rapidly outstripping some of her Northern sisters of greater pretension but less action.

